# Pacemaker and Plateau Potentials Shape Output of a Developing Locomotor Network

**DOI:** 10.1016/j.cub.2012.10.025

**Published:** 2012-12-18

**Authors:** Huaxia Tong, Jonathan Robert McDearmid

**Affiliations:** 1 Department of Biology, College of Medicine, Biological Sciences and Psychology, University of Leicester, Leicester LE1 7RH, UK

## Abstract

**Background:**

During development, spinal networks undergo an intense period of maturation in which immature forms of motor behavior are observed. Such behaviors are transient, giving way to more mature activity as development proceeds. The processes governing age-specific transitions in motor behavior are not fully understood.

**Results:**

Using in vivo patch clamp electrophysiology, we have characterized ionic conductances and firing patterns of developing zebrafish spinal neurons. We find that a kernel of spinal interneurons, the ipsilateral caudal (IC) cells, generate inherent bursting activity that depends upon a persistent sodium current (*I*_*NaP*_). We further show that developmental transitions in motor behavior are accompanied by changes in IC cell bursting: during early life, these cells generate low frequency membrane oscillations that likely drive “coiling,” an immature form of motor output. As fish mature to swimming stages, IC cells switch to a sustained mode of bursting that permits generation of high-frequency oscillations during locomotion. Finally, we find that perturbation of IC cell bursting disrupts motor output at both coiling and swimming stages.

**Conclusions:**

Our results suggest that neurons with unique bursting characteristics are a fundamental component of developing motor networks. During development, these may shape network output and promote stage-specific reconfigurations in motor behavior.

## Highlights

► IC cells of the developing zebrafish CPG exhibit *I*_*NaP*_-dependent bursting activity ► IC cell bursting activity is stage specific ► At coiling stages, IC cells generate rhythmic membrane oscillations ► At swimming stages, IC cells generate high-frequency, sustained bursting

## Introduction

Spinal locomotor circuits are dedicated to the generation of rhythmic activity patterns that underpin coordinated contraction of functionally related muscle groups. These circuits, termed “central pattern generators” (CPGs) form during early development and progress through major functional transitions before generating mature activity [1]. Such transitions are underpinned by maturation of cellular and synaptic properties. However, the role cellular properties play in reconfiguring network output is not fully understood.

Zebrafish are a powerful tool for studying motor ontogeny because their spinal networks are functionally similar to those of mammals yet anatomically simpler and tractable to in vivo analysis [2]. During development, the zebrafish CPG undergoes a series of functional transitions [2] that begin with “coiling” [3], a transient behavior characterized by periodic flexions of the trunk. Coiling is driven by a form of “spontaneous network activity” (SNA) that is commonly observed in developing neural structures [4]. In the zebrafish spinal cord, SNA comprises periodic (0.1–1 Hz), gap junction-synchronized depolarizations that originate from an unknown ensemble of pacemaker currents [5, 6]. Coiling is a relatively short-lived behavior and eventually becomes replaced with “burst swimming” [7, 8]. Characterized by rapid (7–100 Hz) alternating trunk contractions that are driven by patterned neurotransmitter release [3, 7, 8], burst swimming bears no resemblance to coiling and instead serves as a template for adult locomotion [9].

Here we use in vivo electrophysiology to determine how cellular properties influence maturation of zebrafish motor behavior. We show that a kernel of interneurons exhibit intrinsic bursting characteristics that depend upon a persistent sodium current (*I*_*NaP*_). This current mediates bursting in a range of neural networks [10–14], and in a subset of zebrafish interneurons it promotes generation of slow oscillations during coiling and high-frequency bursting during swimming. Because block of *I*_*NaP*_ perturbs both behaviors, we propose that stage-specific bursting is critical to expression of early motor activities.

## Results

### Characterization of Neuron Classes and Network Activity

**Figure 1:**
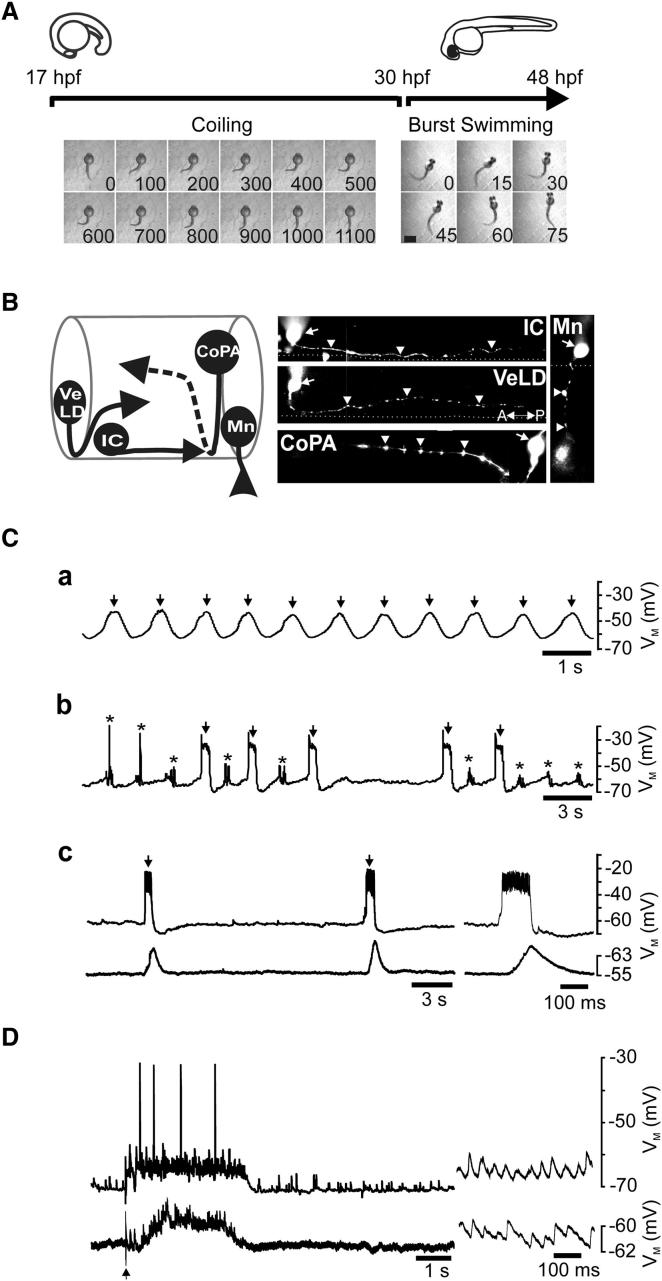
Spinal Neuron Characteristics during the Coiling to Burst Swimming Developmental Period (A) Timeline depicting developmental period encompassing coiling (17–29 hpf) to burst swimming (∼30-48 hpf). Lower panels: consecutive frames of a single coil (left) and three cycles of burst swimming (right). Time (in ms) is shown in bottom right of each frame. A single coil lasts ∼1 s, whereas a single swim cycles lasts ∼30 msec. The scale bar represents 0.5 mm. (B) Schematic (left) and micrographs (right) of primary neuron classes that participate in SNA. Ipsilateral caudal (IC) somata are found in the caudal hindbrain/rostral spinal cord and extend axons ventrolaterally. Ventrolateral descending (VeLD) somata have axons that course ventrally before turning to descend laterally. Commissural primary ascending (CoPA) interneurons have dorsal somata and axons that cross the commisure to ascend contralaterally (hatched line). Motoneurons (Mns) have ventral somata and axons innervating the muscle. Arrows and arrowheads denote position of soma and axons respectively. A-P, anterior-posterior orientation. (C) Network activity during the coiling period. (a) Early coiling stage neurons generate periodic depolarizations (PDs; arrows). (b) Mid-coiling stages neurons generate PDs (arrows) that intersperse with synaptic bursts (SBs; asterisks). (c) At late coiling stages (upper traces), PDs (arrows) are infrequent. Lower traces show activity in a simultaneously recorded muscle cell, revealing that PDs drive motor output. Right-hand panels: expanded sweeps of the same recording showing a single PD in register with neuromuscular activity. (D) At burst swimming stages, neurons (upper trace) generate synaptic drive for burst swimming. Lower traces show activity in a simultaneously recorded muscle cell, revealing that locomotor drive evokes rhythmic neuromuscular activity. Arrow marks stimulus artifact. Right-hand panels: expanded sweep of the same record showing locomotor related EPSPs during swimming. Traces in (Ca–Cc) were obtained from separate IC cell recordings of 17 hpf, 23 hpf, and 26 hpf fish. Trace in (D) was obtained from a Mn at 42 hpf.

In this study we used in vivo patch clamping to survey neuronal firing properties and network activity across the period spanning coiling (∼17–29 hr postfertilization [hpf]) to burst swimming (30–48 hpf; Figure 1A) [3, 7, 8, 15]. We restricted analysis to a subset of the “primary” neurons, an early developing cell population that forms a simple neural scaffold [2]. We focused specifically on ipsilateral caudal (IC), ventrolateral descending (VeLD), and commissural primary ascending (CoPA) interneurons and motoneurons (Mns; Figure 1B) because these are the only cells active at onset of coiling [5, 6]. Recorded cells were visually identified by inclusion of sulforhodamine in the pipette solution (Figure 1B) [5, 6].

During voltage recordings zebrafish spinal neurons generate stage-specific forms of activity [5–8, 15]. At around 17 hpf SNA is first observed. This comprises “periodic depolarizations” (PDs), rhythmic (∼0.6 Hz) membrane oscillations (Figure 1Ca) that are resistant to block of neurotransmitter receptors [5, 6]. By 20–21 hpf, PDs become interspersed with “synaptic bursts” (SBs, Figure 1Cb) that comprise PD-evoked volleys of glycine released from newly integrated “secondary” neurons [5, 6]. As development proceeds, SNA frequency declines and by 26–29 hpf network events are relatively rare (<0.1 Hz, Figure 1Cc). Thereafter (∼30 hpf), SNA terminates and sensory stimulation now evokes fictive “burst swimming.” This comprises 7–100 Hz rhythmic excitatory postsynaptic potentials (EPSPs) superimposed on a sustained tonic drive (Figure 1D) [7].

### Ionic Conductances Necessary for Generation of Coiling

We began by investigating the ionic conductances necessary for SNA generation by screening effects of ion channel blockers on network activity monitored via whole-cell current clamp [5, 6]. We focused on voltage-gated sodium, calcium, and *I*_*h*_ channels because these have previously been implicated in pacemaking [5, 6, 16].

**Figure 2:**
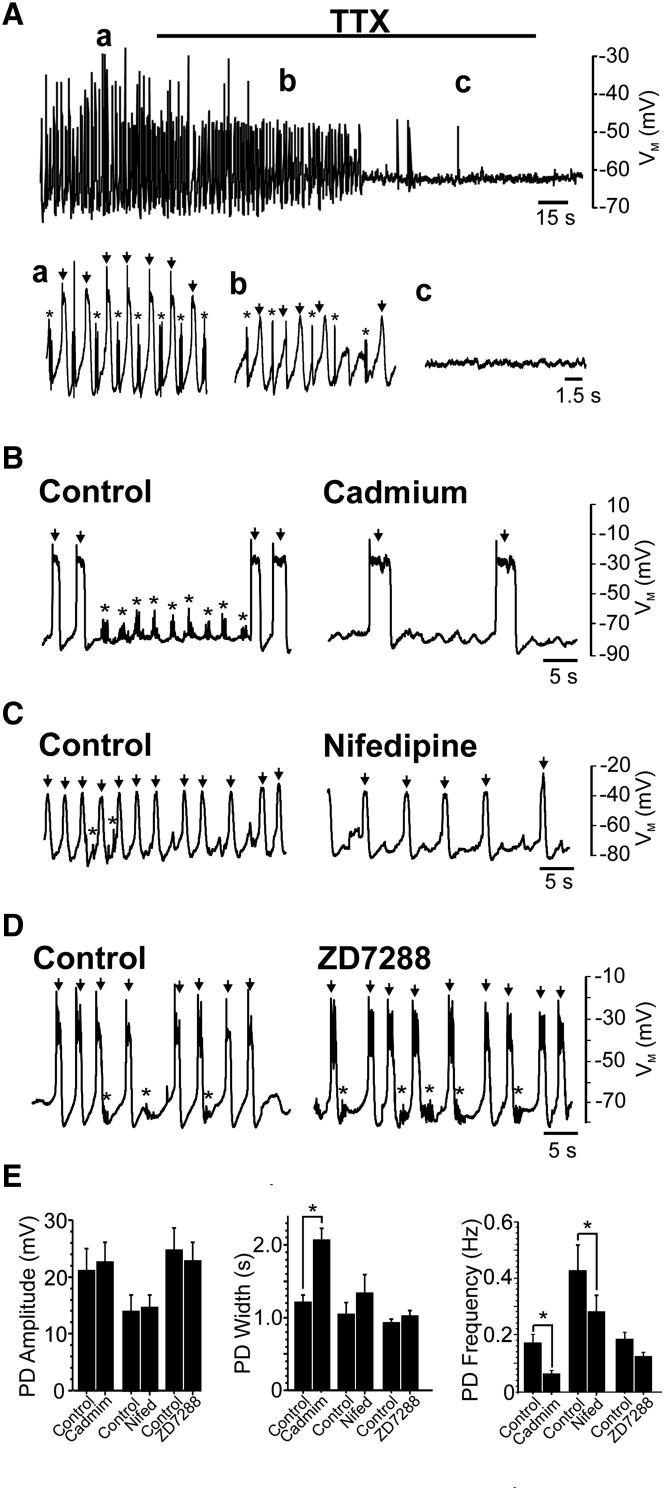
Effects of Ion Channel Blockers on SNA (A) Upper panel: voltage recording depicting effects of TTX (0.5 μM) on SNA. Lower panel: expanded traces of above recording during control (a) and after 1 min (b) and 2.5 min (c) TTX perfusion. PDs (arrows) and SBs (asterisks) were completely abolished by this treatment (c). (B) After 7 min cadmium (100 μM) perfusion, SBs (asterisks) were abolished, whereas PDs (arrows) persisted. Note the presence of low-amplitude oscillations after cadmium treatment, which represent filtered PDs originating from electrically coupled contralateral neurons [5]. (C) Nifedipine (100 μM) reduced SBs but failed to inhibit PDs. (D) ZD7288 (40 μM) blocked neither PDs (arrows) nor SBs (asterisks). (E) Bar charts depicting effects of cadmium, nifedipine and ZD7288 on mean PD amplitude (left-hand panel), width (middle panel), and frequency (right-hand panel). Note that PD frequency was significantly reduced by cadmium (control = 0.18 ± 0.03 Hz, cadmium = 0.06 ± 0.01 Hz; ^∗^p = 1.6 × 10^−3^) and nifedipine (control = 0.43 ± 0.09 Hz, nifedipine = 0.28 ± 0.06 Hz; ^∗^p = 0.02). Cadmium also significantly increased PD widths (control = 1.21 ± 0.74 s, cadmium = 2.07 ± 0.16 s; ^∗^p = 4.7 × 10^−4^). Data are represented as mean ± SEM. See also Figure S1.

As previously reported [5, 6], bath perfusion of the sodium channel blocker tetrodotoxin (TTX, 0.02–1 μM; n = 12) rapidly abolished both PDs and SBs (Figure 2A). In contrast cadmium, a panspecific calcium channel blocker (50–200 μM; n = 15) abolished SBs but not PDs (Figure 2B). However, this treatment markedly increased PD duration and reduced SNA frequency (Figure 2E). The L-type calcium channel blocker nifedipine (50–100 μM, n = 6) attenuated SBs and reduced PD frequency without affecting other SNA parameters (Figures 2C and 2E), whereas the T-type calcium channel antagonist efonidipine (100 μM, n = 5, Figures S1A and S1D available online) and the *I*_*h*_ antagonist ZD7288 (10–50 μM, n = 15, Figures 2D and 2E) had no effect on SNA. This suggests that only sodium channels are necessary for SNA generation.

We next examined currents involved in PD termination. Prior studies suggest that calcium-dependent potassium currents (*I*_*KCa*_) mediate PD repolarization [6] which could underlie the PD-broadening effects of cadmium. To address this, we reduced the extracellular calcium concentration (to 0.5–1 mM). As expected, this treatment doubled PD durations (n = 9, Figures S1B and S1D) but, unexpectedly, increased SNA frequency (Figure S1D). In contrast, block of *I*_*KCa*_ with apamin (20–40 nM, n = 6) prolonged PD widths without affecting other SNA parameters (Figures S1C and S1D). These findings confirm that *I*_*KCa*_ regulates PD repolarization and further suggest that calcium ions depress neuronal excitability.

### I_NaP_ Is Necessary for Coiling Activity

**Figure 3:**
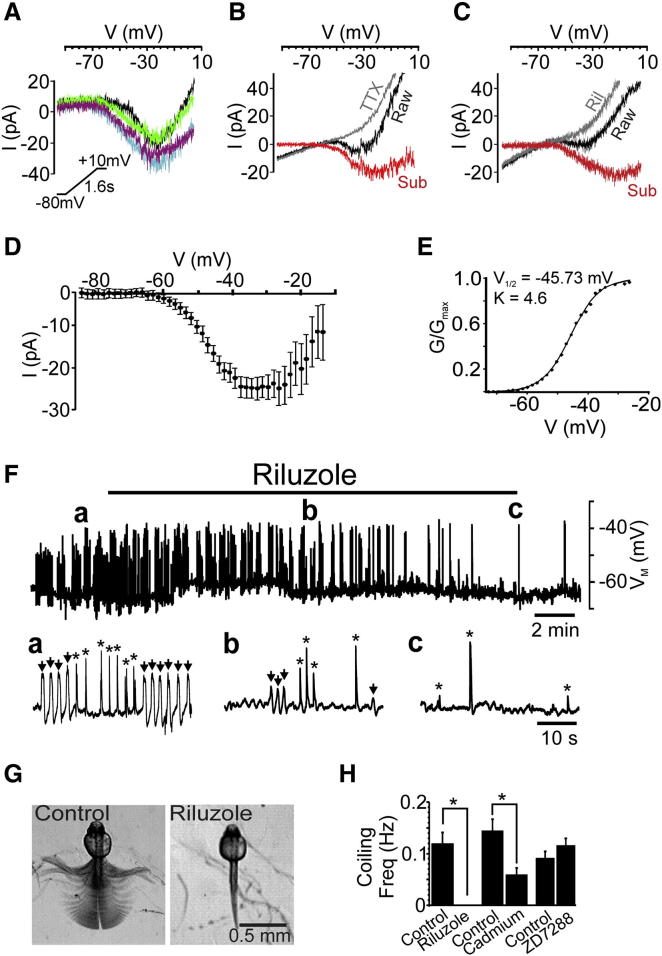
Spinal Neurons Express I_NaP_ during the SNA Period (A) In the presence of cadmium and caesium (see the Supplemental Experimental Procedures) 55 mV/s voltage ramps (inset) generated a persistent inward current in IC (purple), VeLD (blue), CoPA (green), and Mn (black) cells at 24 hpf. All traces are leak subtracted. (B and C) Persistent inward currents (black) were blocked by 1 μM TTX and 5 μM riluzole (gray). Subtraction currents reveal isolated sodium current (red). (D) IV plot of riluzole-sensitive current (mean of 14 neurons). (E) Boltzmann fit of riluzole sensitive conductance. V_1/2_, half activation voltage; K, Boltzmann constant. (F) Effects of riluzole (5 μM) on SNA recorded from an IC cell at 23 hpf. The lower panel shows expanded regions of activity in control (a) and after 8 min (b) and 20 min (c) riluzole. Arrows and asterisks show PD and SB, respectively. (G) Overlaid frames (100 ms intervals) of behavior in control and after 5 μM riluzole treatment. (H) Bar chart of average coiling frequency. Riluzole (5 μM) abolished coiling behavior (control n = 10, riluzole = 15; ^∗^p = 3.1 × 10^−7^), whereas 100 μM cadmium reduced coiling frequency (control = 12, cadmium n = 11; ^∗^p = 0.005) and 200 μM ZD7288 (control n = 14, ZD7288 n = 16; p = 0.2) had no effect. Data are represented as mean ± SEM. See also Figure S2.

We reasoned that *I*_*NaP,*_ a sodium current often implicated in pacemaking [10–14], might underpin SNA. To test for the presence of this current, we voltage clamped neurons under conditions designed to minimize contamination from calcium and potassium channels. We then applied slow voltage ramps to inactivate transient sodium currents (*I*_*NaT*_), thereby permitting isolation of *I*_*NaP*_. In ohmic regions of the ramp (around −80 mV) the V-I relationship corresponded to neuronal input resistance (IC = 1.82 ± 0.18 GΩ, n = 17; VeLD = 1.88 ± 0.19 GΩ, n = 8; CoPA = 2.39 ± 0.20 GΩ, n = 17; Mn = 1.73 ± 0.10 GΩ, n = 22). As ramps approached −65 mV, all cell types exhibited inward rectification (Figure 3A). This was mediated by sodium because it could be blocked by tetrodotoxin (TTX; 0.5–1 μM, Figure 3B) and riluzole (5 μM, Figure 3C). Isolation of this current, by subtracting the riluzole-blocked current from the control current (n = 14), revealed subthreshold activation at −66.21 ± 1.04 mV (spike threshold = −42.0 ± 1.14 mV, n = 20) and a peak amplitude of −23.66 ± 2.53 pA (Figure 3D). A Boltzmann fit of this current revealed a half-activation of −45.73 mV (Figure 3E). These data suggest that primary neurons of the coiling circuit express a persistent sodium current (hereafter referred to as “*I*_*NaP*_”).

There are few selective inhibitors of *I*_*NaP*_; most drugs also block *I*_*NaT*_. However, riluzole is reported to exhibit selectivity for *I*_*NaP*_ at low micromolar concentrations [14, 17]. To determine whether riluzole affects *I*_*NaT*_ we recorded from Mns at 20–24 hpf, a stage when these cells typically fire unitary or paired spikes (Figure S2A). At 5 μM, a dose sufficient to inhibit *I*_*NaP*_ (Figure 3C), riluzole did not affect spike amplitude or rheobase (Figures S2B and S2C). However, ≥10 μM riluzole reduced spike amplitude (Figure S2B). We thus assessed the impact of 5 μM riluzole on SNA. This treatment gradually reduced PD amplitudes until they were abolished (n = 11, Figure 3F). A coincident loss of SBs was observed, likely because PDs trigger these events [5]. Similarly, riluzole (5 μM) blocked coiling behavior in freely moving embryos, whereas injection with cadmium (100 μM) or ZD7288 (200 μM) did not (Figures 3G and 3H). Taken together, these findings suggest that *I*_*NaP*_ drives coiling activity.

### Inherent I_NaP_-Dependent Bursting Activity at Coiling Stages

**Figure 4:**
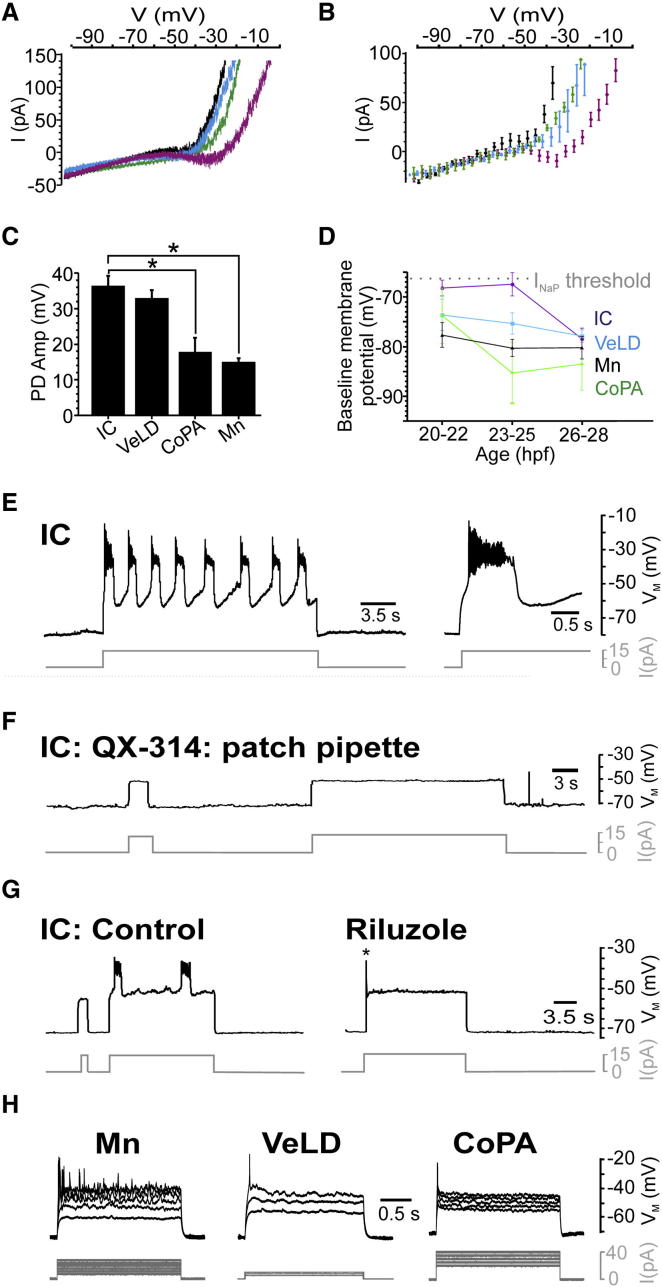
IC Cells Have Unique Cellular Characteristics during Coiling Stages (A and B) Raw traces (A) and mean current responses (B) to 55mV/s voltage ramps in the absence of calcium and potassium channel blockers (see the Supplemental Experimental Procedures). IC cells (purple) exhibited inward rectification, whereas VeLD (blue), CoPA (green), and Mn (black) cells did not. (C) Mean PD amplitude (amp) did not differ between IC and VeLD neurons (IC = 36.41 ± 2.77 mV, n = 31; VeLD = 32.86 ± 2.30, n = 22, p = 0.43) but was significantly smaller in CoPA and Mn cells (CoPA = 17.86 ± 4.00, n = 13, p = 0.002; Mn = 14.98 ± 1.12, n = 26, p = 1.87 × 10^−9^). (D) Mean baseline membrane potential of IC cells was not significantly different from *I*_*NaP*_ threshold (−66.21 ± 1.04 mV, hatched gray line) at 20–22 hpf (mean = −68.13 ± 1.60 mV, n = 7, p = 0.31) and 23–25 hpf (mean = −67.37 ± 2.33 mV, n = 9, p = 0.61) but was highly significantly different at 26–28 hpf (mean = −78.06 ± 2.10, n = 19, p = 1 × 10^−4^). Baseline membrane potential of VeLD (20–22 hpf, n = 6; 23–25 hpf, n = 7; 26–28 hpf, n = 18), CoPA (20–22 hpf, n = 4; 23–25 hpf, n = 6; 26–28 hpf, n = 7), and Mn (20–22 hpf, n = 16; 23–25 hpf, n = 6; 26–28 hpf, n = 15) cells was significantly different from *I*_*NaP*_ threshold at all developmental stages (p < 0.05). (E) Left-hand panel: representative voltage traces showing IC cell membrane oscillations evoked by current injection. Right-hand panel: expanded sweep of the same record showing onset of oscillatory response. (F) On dialysis with QX-314 (4 mM), IC cells were unable to generate membrane oscillations or action potentials when depolarized. (G) IC cell responses in control and after 4 min bath perfusion with riluzole (5 μM). Riluzole abolished oscillations, but cells still fired single action potentials (asterisk) in response to depolarization. (H) Membrane responses of VeLD, CoPA, and Mns to a family of current pulses. These cells generated single or tonic action potential discharges at suprathreshold potentials. Traces in (E–H) are derived from separate current clamp recordings spanning 26–28 hpf. Data in (C) and (D) represented as mean ± SEM. Current commands are depicted by gray traces. See also Figure S3.

Previously, we had isolated *I*_*NaP*_ by blocking calcium and potassium currents that may have occluded it. However, if *I*_*NaP*_ acts as a pacemaker current during SNA, it should be detectable under normal physiological conditions. To address this issue, we monitored V-I profiles in the absence of ion channel blockers. These differed between cell types (Figures 4A and 4B). IC cells exhibited TTX-sensitive (Figure S3A) inward rectification (n = 7). In contrast, inward rectification was not observed in VeLD (n = 5), CoPA (n = 5), and Mn (n = 4) cells, although subsequent addition of TTX revealed its presence (Figure S3B). This suggests *I*_*NaP*_ is the predominant subthreshold current of IC cells, whereas it is masked by outward rectification in other cells.

Voltage recordings of network activity revealed other cell-specific differences. IC and VeLD interneurons generated robust, large amplitude PDs, whereas CoPA and Mn PDs were significantly smaller (Figures 4C and S3C). Moreover, baseline membrane potential (membrane potential during inactive periods) of 20–25 hpf IC cells did not differ significantly from *I*_*NaP*_ threshold, suggesting that this current is engaged at rest (although baseline membrane potential hyperpolarized at later stages). In other cell types, baseline membrane potential was negative to *I*_*NaP*_ threshold, irrespective of age (Figure 4D).

The above observations suggested IC cells may be central to SNA generation. We thus examined their cellular properties in detail. To this end, we current clamped IC cells at 26–29 hpf, a period when PDs are infrequent (≤0.1 Hz) and cellular properties more easily studied (see the Supplemental Experimental Procedures). Remarkably, subthreshold depolarization (mean threshold = −51.17 ± 1.29 mV) triggered robust voltage oscillations in IC cells (n = 10, Figure 4E). These closely resembled SNA, being characterized by repetitive 1.19 ± 0.09 s depolarizations that attained a plateau voltage of −28.84 ± 2.15 mV upon which small spikelets occurred. Oscillations typically persisted for the duration of the stimulus but never outlasted it (Figure S3D), indicating a strong voltage dependence. Oscillation frequency also varied with current magnitude, a typical feature of pacemaker cells (Figure S3E).

To determine whether oscillations were sodium dependent, we dialyzed IC cells with QX-314, a cell-impermeant sodium channel blocker (4 mM, Figure 4F). This abolished membrane oscillations and spike capability (n = 4 of 4). Bath perfusion with riluzole (5 μM) also abolished membrane oscillations, though neurons still fired single spikes upon depolarization (n = 6, Figure 4G). Importantly, oscillations were not observed in other primary neurons, which invariably generated single (VeLD, n = 13; CoPA, n = 13) or tonic (Mn, n = 16) spike discharges when depolarized (Figure 4H). These data suggest that coiling stage IC cells possess *I*_*NaP*_-dependent pacemaker properties.

### Inherent I_NaP_-Dependent Bursting at Onset of Swimming

On transition to swimming, the spinal CPG undergoes a major functional reconfiguration. Chemical synaptogenesis and secondary neuron integration undoubtedly contribute to this event. However, maturation of neuronal firing properties may also play a role. We therefore asked how IC cell properties change during this transition.

**Figure 5:**
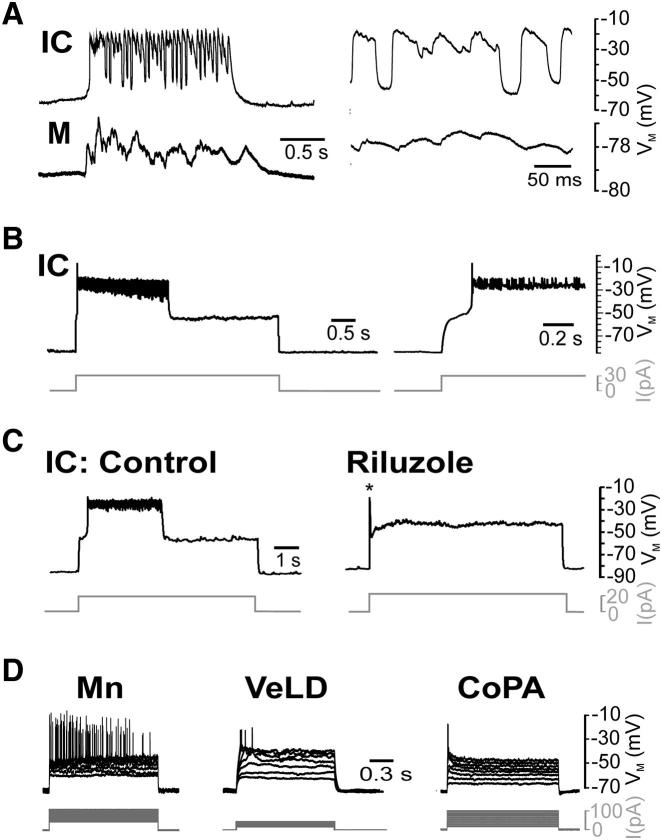
IC Cells Generate I_NaP_-Dependent Sustained Bursts at the Early Swimming Stage (A) Left-hand panel: simultaneous voltage recordings of an IC and red muscle (M) cell during fictive swimming in a 35 hpf embryo. Right-hand panel: expanded region of the same record showing rhythmic bursting activity occurring in register with neuromuscular drive. (B) Left-hand panel: voltage recording of IC cell response to current injection. Current injection evoked a sustained burst (in this case lasting 2.8 s). Right-hand panel: expanded region of the same record showing step-like depolarization at burst onset. (C) Five minute perfusion with riluzole (5 μM) abolished sustained bursting. Note that cells were able to fire single action potentials (asterisk) in the presence of riluzole. (D) Voltage responses of non-bursting primary neurons to a family of current pulses. Upward deflections are tonic (Mn) or single (VeLD, CoPA) action potential discharges. Traces in (A–D) are derived from separate current clamp recordings spanning 32–46 hpf. Current commands are depicted by gray traces. See also Figure S4.

We first asked whether IC cells integrate into the swimming CPG. Simultaneous IC cell-muscle recordings confirmed that they do, generating rhythmic membrane depolarizations in register with locomotor activity (Figure 5A, n = 3). We therefore examined intrinsic properties of IC cells at early swimming stages (30–48 hpf). Here, current injection triggered a sustained, step-like jump in membrane potential upon which small amplitude spikelets (mean frequency = 51.49 ± 6.14 Hz) occurred (Figure 5B, n = 18). These “sustained bursts” activated at a threshold of −56.37 ± 1.93 mV and attained a plateau voltage of −35.19 ± 2.09 mV. Their duration varied from 1 s to >20 s (mean duration = 4.87 ± 1.07 s), and they typically terminated spontaneously, although bursts were sometimes followed by shorter events (Figure S4A). However, release of current invariably terminated bursting (Figure S4B), suggestive of strong voltage dependence.

We next asked whether sustained bursting was also mediated by *I*_*NaP*_. Perfusion of TTX (1 μM, n = 5; Figure S4C) or riluzole (5 μM, n = 12, Figure 5C) blocked sustained bursts, although riluzole-treated cells remained capable of generating single spikes when depolarized. In contrast, bursting was never observed in VeLD (n = 21), CoPA (n = 6), or Mns (n = 14), which generated fast spikes on depolarization (Figure 5D). Thus, on transition to swimming, IC cells switch from an *I*_*NaP*_-dependent oscillatory to an *I*_*NaP*_-dependent burst mode of firing.

### I_NaP_-Dependent Bursting Activity and Locomotion

**Figure 6:**
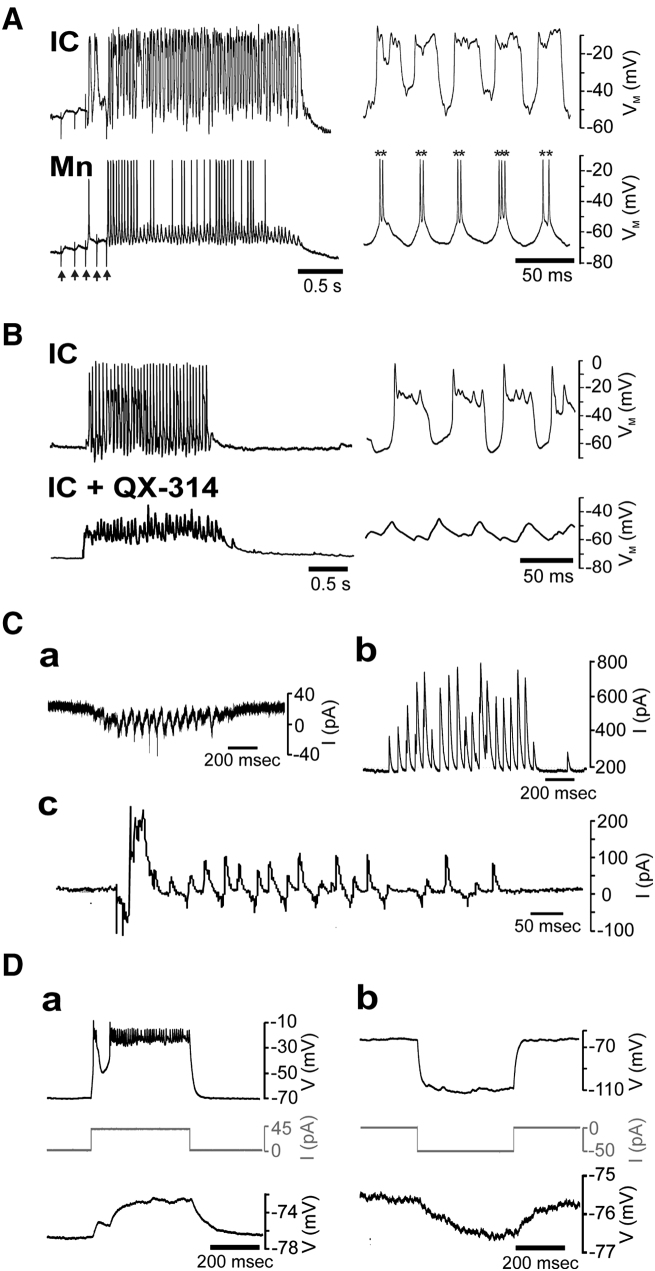
Locomotor-Related Activity in IC cells (A) Left-hand panels: paired voltage recording of locomotor activity recorded from an IC cell and Mn at 38 hpf. Right-hand panel: expanded sweep taken from same episode showing rhythmic IC cell bursting occurring in register with rhythmic Mn EPSPs. Note transient spikes (asterisks) often occurred at peak of Mn EPSPs. Arrows mark stimulus artifacts. (B) Left-hand panels: representative IC cell locomotor drive recorded with control intracellular solution (upper trace) and intracellular solution containing 4 mM QX-314 (lower trace). Right-hand panels: expanded excerpts of the same locomotor episodes. Note that large amplitude rhythmic bursts are not observed in QX-314 dialyzed cells. Recordings were obtained from embryos at 35 hpf (control) and 37 hpf (QX-314), respectively. (C) Representative voltage clamp recordings of locomotor-related drive in 33–35 hpf IC cells. At the estimated chloride ion reversal potential (−43 mV), the cell received rhythmic inward currents superimposed upon a small sustained inward current (a). At the estimated cation reversal potential (7 mV), the same cell received outward currents (b). At an intermediate holding potential (−23 mV), rhythmic inward currents interspersed with volleys of outward current were observed (c). (D) Simultaneous voltage recordings of electrical coupling between an IC cell (upper trace) and a Mn (lower trace). Depolarization of IC cells causes tonic depolarization of the coupled Mn (a). Similarly, injection of IC cells (upper trace) with hyperpolarizing current steps evokes hyperpolarizing responses in the coupled Mn (lower trace) (b).Traces in (a) and (b) were obtained from embryos at 30 and 34 hpf, respectively. Current commands are depicted by gray traces. See also Figure S5.

We next performed simultaneous recordings to compare locomotor activity of IC cells with that of ipsilateral VeLDs (n = 3) and Mns (n = 5). Although IC cells were always coactive with these cells (Figures 6A and S5A), their activity patterns differed. Specifically, IC cells generated atypically large amplitude (30.19 ± 1.14 mV), prolonged (53.23 ± 3.38 ms), rhythmic membrane depolarizations upon which small amplitude spikelets were observed (Figure S5A). This contrasted with Mn and VeLD activity patterns, where brief, large-amplitude spikes were triggered by comparatively low-amplitude EPSPs (Figures 6A and S5A).

To determine whether *I*_*NaP*_ drives the unique activity patterns of IC cells, we first dialyzed recorded neurons with QX-314 (4 mM, n = 3). This abolished both sustained bursts and action potentials (data not shown) and profoundly affected locomotor drive (Figure 6B). The large, sustained depolarizations typical of IC cells were no longer seen, being replaced by low amplitude (mean = 15.47 ± 0.40 mV) depolarizations of similar frequency (20.80 ± 0.80 Hz). To visualize *I*_*NaP*_ fluxes during simulated locomotion, we used a variant of the action potential clamp technique [18]. Here episodes of locomotor activity, obtained from IC cell voltage recordings, were used as command waveforms during voltage clamp (Figure S5Ba). The sodium component of the current response (isolated by subtraction of TTX-blocked currents from control currents) comprised cyclically active persistent inward currents (mean amplitude = 60.80 ± 13.47 pA) that flowed during depolarizing phases of the voltage command (n = 5, Figure S5Bb). Often, *I*_*NaT*_ occurred at the peak of the persistent inward currents (Figure S5Bb). Collectively these findings suggest that the locomotor-related bursting observed in IC cells depends upon activation of *I*_*NaP*_.

The residual locomotor drive observed in QX-314-treated IC cells suggested that they might receive extrinsic synaptic input. Other CPG neurons of the zebrafish spinal cord receive glutamatergic and glycinergic locomotor drive [7]. These components can be isolated by voltage clamping at the reversal potential for chloride ions (to isolate the glutamatergic cationic conductance) and cations (to isolate the glycinergic chloride current) [7]. When clamped at the chloride reversal potential, IC cells generated inward currents during locomotion (Figure 6Ca, n = 8). These comprised rhythmic (23.81 ± 1.87 Hz) and tonic components that were abolished by kynurenic acid, a glutamate receptor antagonist (n = 3, data not shown). In contrast, clamping at the cation reversal potential revealed the presence of outward currents (Figure 6Cb, n = 10) that were blocked by strychnine, a glycine receptor antagonist (n = 3, data not shown). Clamping at intermediate potentials revealed alternation between rhythmic inward (presumably glutamatergic) and outward (presumably glycinergic) currents (Figure 6Cc, n = 10). Thus, IC cells receive glutamatergic and glycinergic inputs during locomotion.

We also examined synaptic targets of IC cells during simultaneous recording of Mns (n = 15), VeLDs (n = 4), or IC cells (n = 1). In 18 of 20 paired recordings, clear evidence of coupling was observed (in two Mn recordings coupling was not seen). Here, IC cell bursts evoked sustained 2.08 ± 0.36 mV depolarizations in the paired neuron (Figure 6Da). Postsynaptic responses did not appear to be mediated by chemical synapses because we observed no evidence of EPSPs and responses were unaffected by application of kynurenic acid (2–4 mM) and strychnine (1 μM; n = 12, data not shown). Thus, we asked whether they were gap junction dependent. Negative current commands evoked hyperpolarizing responses in the coupled cell, confirming that this was the case (Figure 6Db). Estimates of the coupling coefficient (voltage change in postsynaptic cell as a percent of voltage change in IC cell) yielded a value of 4.63% ± 0.78%. These observations suggest IC cells of the early swimming network are electrically coupled to CPG neurons.

### Possible Functions of I_NaP_-Dependent Bursting during Locomotion

**Figure 7:**
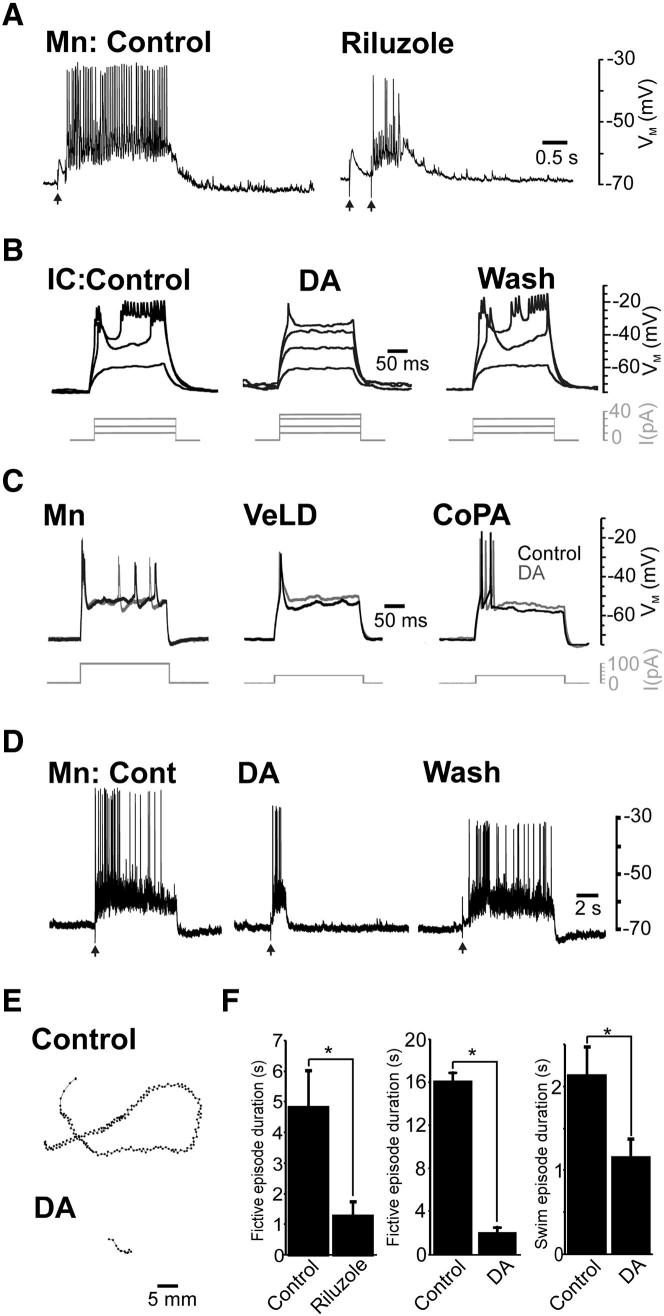
Possible Roles of IC Cells during Swimming (A) Locomotor-related activity recorded from a 37 hpf primary Mn in control saline and after 10 min incubation in riluzole (5 μM). Arrows mark stimulus artifacts. (B) Effects of dopamine (DA) on IC cell firing at 30–48 hpf. Sustained bursts (left-hand trace) were abolished by 5 min perfusion with 10 μM DA (middle trace) but recovered after 5 min wash (right-hand trace). Current commands are depicted by gray traces. (C) Dopamine (DA) did not affect action potential firing in other neurons. Current commands are depicted by gray traces. (D) Example of locomotor-related drive recorded from Mns exposed to control saline, after 5 min dopamine (DA) perfusion and after 10 min wash. Arrows mark stimulus artifacts. (E) Plot of example swim trajectories of control and dopamine-treated fish at 42 hpf. (F) Bar charts depicting effects of riluzole and dopamine (DA) on the duration of Mn fictive episode duration (left-hand and middle graphs) and swim episode length (right-hand graph). Riluzole reduced the duration of locomotor drive from 4.84 ± 1.17 s to 1.29 ± 0.42 (^∗^p = 1.8 × 10^−3^). Similarly dopamine reduced locomotor drive duration from 16.14 ± 0.76 s to 2.17 ± 0.39 s (^∗^p = 9 × 10^−3^) and swim duration from 2.14 ± 0.33 s to 1.17 ± 0.20 s, ^∗^p = 0.01). Data are represented as mean ± SEM. See also Figure S6.

Finally, we asked whether disruption of IC cell bursting affected locomotor drive, as monitored via voltage recordings of 35–40 hpf Mns. Acute (10 min) exposure to 5 μM riluzole reduced locomotor episode durations by 73% (n = 8, Figures 7A and 7F) without affecting EPSP frequency (Figure S6A), whereas prolonged (30–60 min) exposure completely abolished locomotor output (data not shown). However, because riluzole also blocked repetitive Mn spiking in swimming stage fish (n = 8, Figure S6B), these effects may not be solely due to impairment of IC cell bursting. We thus sought a more specific method of inhibiting IC cells. During a prior neuromodulator screen, we found that dopamine suppresses IC cell firing. We therefore characterized effects of dopamine on the four primary neuron classes studied. During IC cell voltage recordings at 35–48 hpf (n = 8), dopamine perturbed sustained bursting (Figure 7B), increased spike threshold by 46% (Figure S6C), and hyperpolarized resting membrane potential by 6.71 ± 0.97 mV (Figure S6C). In contrast, dopamine had no effect on other primary neurons, except for VeLDs (n = 6), where spike threshold significantly decreased (Figures 7C, S6C, and S6D). These data suggest dopamine can be used to selectively inhibit IC cells.

The effects of DA on locomotor drive were examined during whole-cell Mn recordings of 40–44 hpf fish. Like riluzole, dopamine decreased motor episode length (by 84%, Figure 7D and 7F) without affecting EPSP frequency (Figure S6E). Similar effects were observed during locomotor behavior experiments, with dopamine-injected (100 μM, n = 26) fish exhibiting a 46% reduction in swim episode duration when compared to controls (n = 26, Figure 7E and 7F). These findings suggest that IC cell bursting is necessary for maintenance of swimming activity.

Finally, we asked whether hyperpolarization-mediated silencing of IC cells could mimic the effects of dopamine. During voltage recordings, strong hyperpolarization of IC cells had no effect on locomotor drive of simultaneously recorded Mns or muscle fibers (n = 3, Figure S6F). Thus, inhibition of individual IC cells is not sufficient to impair network activity.

## Discussion

Under conditions of enhanced background excitation or ion channel modulation, vertebrate spinal neurons can generate membrane oscillations and plateau potentials [13, 19–27], and in some instances *I*_*NaP*_ contributes to this phenomenon [13, 25, 26]. However, the significance of *I*_*NaP*_ to motor maturation is not fully understood. Here we show that this current serves key roles during zebrafish CPG development, first generating SNA-like oscillations during coiling and subsequently generating high-frequency, rhythmic bursts during swimming. Our findings suggest that neurons with *I*_*NaP*_–dependent bursting characteristics facilitate stage-specific reconfigurations in motor output.

### I_NaP_-Dependent Bursting in the Network for SNA

Previous studies [5, 6] have shown that TTX blocks SNA in the zebrafish spinal cord, suggesting a role for sodium conductances. Here we extend these observations to show that *I*_*NaP*_ may be essential for expression of SNA. Evidence for this stems from the observation that embryonic neurons express *I*_*NaP*_ and that riluzole-mediated block of this current abolishes network output. In contrast, neither *I*_*h*_ nor calcium channels are critical for SNA generation, although the latter is known to influence PD repolarization (via *IK*_*Ca*_ activation [6]), and our data suggest that it also affects SNA frequency (possibly via *I*_*NaP*_ modulation [13, 28]). However, before *I*_*NaP*_ can be implicated in SNA generation, potential off-target effects must be considered. Aside from its actions on *I*_*NaP*_, riluzole is known to inhibit chemical synapses [29, 30] and calcium [31], potassium [32, 33], and transient sodium channels [34–36]. Nonetheless, such effects are unlikely to account for our observations because they occur at higher concentrations than used in this study (>10 μM). Moreover, block of synaptic transmission [6], calcium channels (current study), or potassium channels (J.R.M., unpublished data) does not abolish SNA. Finally, at the concentration used, riluzole did not affect action potential waveforms, and because coiling stage neurons predominantly fired single spikes, an effect on spike adaptation is unlikely. In sum, these observations strongly suggest that riluzole impairs SNA through direct block of *I*_*NaP*_.

Our data provide evidence for the cellular origins of SNA. Current clamp experiments revealed that IC cells uniquely generate *I*_*NaP*_-dependent membrane oscillations that are indistinguishable from SNA. This suggests that a pacemaker kernel drives activity in this immature, electrically coupled network. Although this contrasts with the majority of embryonic networks in which SNA is thought to arise from recurrent synaptic excitation [4], recent work suggests that pacemaker neurons are often embedded within these circuits [12, 37–39]. Thus, *I*_*NaP*_-dependent pacemaker activity may be a common feature of SNA circuitry, irrespective of whether activity propagates via electrical or chemical synapses.

As *I*_*NaP*_ is expressed in both bursting and nonbursting neurons, pacemaker potential must be determined by additional cellular properties. In rodent Pre-Bötzinger [40] and developing dorsal horn neurons [12], the balance between *I*_*NaP*_ and resting potassium currents determines oscillatory potential. Our data indicate a similar scenario in zebrafish because under physiological conditions IC cells exhibit inward rectification at subthreshold voltages whereas nonoscillatory neurons do not. Another characteristic of IC cells is their depolarized membrane potential. During early coiling periods, membrane potential lies close to *I*_*NaP*_ threshold, and these cells are thus likely to oscillate autonomously. The gradual membrane hyperpolarization that occurs as embryos age might contribute to the progressive slowing of SNA frequency [3, 5].

### I_NaP_-Dependent Bursting in the Swimming CPG

At the onset of swimming, IC cells generate sustained forms of bursting that also depend upon *I*_*NaP*_. Such bursting superficially resembles plateau potentials reported in spinal neurons of other vertebrates [19, 20], although closer analysis reveals key differences. First, plateau potentials outlast the depolarizing stimulus that triggered them. Second, calcium conductances drive plateau potentials (but see [25]). Third, plateau potentials are neuromodulator dependent. Thus, the activity we report is, to our knowledge, unique. Although the mechanisms responsible for the switch to sustained bursting are not known, they may arise from changes in *I*_*NaP*_ inactivation kinetics or downregulation of slowly developing potassium conductance (such as *I*_*KCa*_ [6]).

In comparison to the other studied neurons, IC cells generate unique locomotor activity patterns that comprise atypically large, prolonged rhythmic bursts. Such activity appears to arise from the intrinsic properties of these cells, which generate cyclical *I*_*NaP*_-dependent bursts in response to rhythmic synaptic input. At early swimming stages, IC cells form electrical synapses with downstream CPG elements and electrotonic spread of bursting likely amplifies the locomotor drive. This could be critical for generation of motor activity at stages when insufficient chemical synapses have formed. In support of this hypothesis, we found that motor episodes cannot be maintained when IC cell bursting is blocked (with dopamine). Thus, the unique bursting properties of IC cells may be fundamentally important to motor generation.

### Conclusions

To conclude, our study suggests that spinal neurons with *I*_*NaP*_-dependent bursting properties may be central to generation of early motor behaviors. These findings represent an important step forward in defining the role of intrinsic bursting within emerging motor networks and further suggest that developmental changes in bursting facilitate reconfigurations in CPG output.

## Experimental Procedures

All experiments were conducted in accordance with the Animals (Scientific Procedures) Act 1986. Full experimental details are provided in the Supplemental Experimental Procedures.

## Supplemental Information

Document S1. Supplemental Experimental Procedures and Figures S1–S6
