# Supplementary material for: Pacemaker and Plateau Potentials Shape Output of a Developing Locomotor Network
Source: Curr Biol. 2012 Dec 18;22(24):2285–93. doi: 10.1016/j.cub.2012.10.025 (PMC3525839; doi:10.1016/j.cub.2012.10.025)
Supplement: Document S1. Supplemental Experimental Procedures and Figures S1–S6 [file mmc1.pdf]

Current Biology, Volume *22*

Supplemental Information

Pacemaker and Plateau Potentials Shape Output of a Developing Locomotor Network

Huaxia Tong and Jonathan Robert McDearmid

SUPPLEMENTAL EXPERIMENTAL PROCEDURES

Animals and preparation for in vivo recording

Zebrafish were maintained according to established procedures [1] and all experiments carried out in compliance with the Animals (Scientific Procedures) Act, 1986. Embryo staging was performed in accordance with Kimmel *et al*. [2] and animals were prepared for patch clamp electrophysiology as previously described [3]. Briefly, embryos or larvae were anesthetised and pinned into a Sylgard-lined dish containing 0.02-0.04% tricaine (MS-222) dissolved in Evans solution (constituents in mM: 134 NaCl, 2.9 KCl, 2.1 $CaCl_2$, 1.2 $MgCl_2$, 10 HEPES, and 10 glucose, osmolarity 280–290 mosM, pH 7.8 adjusted with NaOH). The skin overlying myotomal tissue was removed with fine forceps. In most experiments, tricaine was subsequently replaced with Evans containing the neuromuscular blocker D-tubocurarine (10 µM) to ensure muscle paralysis during experiments. However, during recordings of neuromuscular activity, embryos were partially paralysed via exposure to reduced D-tubocurarine concentrations (3 µM). Fish were then transferred to the patch clamp microscope (Nikon FN-1) and muscle fibres removed from a two to four somite region via aspiration with a broken patch pipette. Recordings were made between somites 4 to 9 as rostral spinal regions have previously been shown to be critical for generation of SNA.

Patch clamp electrophysiology

Patch pipettes were pulled from filamented borosilicate glass (GC150F-10, Harvard Apparatus, Edenbridge, UK) using a P-80 micropipette puller (Sutter Instrument, Novato, CA, USA). Pipettes with resistance ranging between 3-5 MΩ and 3-10 MΩ were used for voltage and current clamp recordings respectively. Depending on the experiment, pipettes were filled with one of three recording solutions: a potassium-based internal solution, containing (mM): K-Gluconate 126, KCl 6, NaCl 10, HEPES 10, EGTA 10, $MgCl_2$ 2, pH to 7.2); a caesium-based recording solution, where K-gluconate (126 mM) and KCl (6 mM) were substituted with CsCl (132 mM) or; when examining locomotor drive, a low chloride solution containing (mM): 125 K-gluconate, 2.5 $MgCl_2$, 10 EGTA, 10 HEPES. In voltage clamp and some, but not all, current clamp experiments 1 mM Mg-ATP and 0.1 mM Na-GTP were included in the patch pipette. As these reagents had no detectable effects on physiological properties of embryonic neurons, data was pooled with experiments in which these reagents were not included. For experiments in which sodium channels were blocked in the recorded cell, QX-314 (4 mM) was added to the pipette solution. Sulforhodamine B was routinely included in the patch pipette solution to facilitate visualization of neurons under fluorescence. Neurons were classified in accordance with previously established morphological criteria [4-7].

Recordings were amplified with a Multiclamp 700B (Molecular Devices, Sunnyvale, CA, USA) or an RK-400 (BioLogic, Claix, France) amplifier. Data were digitised with an Axon Digidata 1440 A (Molecular Devices, Sunnyvale, CA, USA) A-D converter connected to a PC running either pClamp 10 (Molecular Devices) or WinEDR (http://spider.science.strath.ac.uk/sipbs/software_ses.htm). Raw signals were acquired at 10-20kHz and low-pass filtered at 5-10kHz. For voltage-clamp, series resistance was routinely compensated by 70-80%. All traces were compensated for a junction potential of −13.7 mV.

During initial voltage-current (V-I) experiments in which I_{NaP} was studied we used conditions designed to minimize contamination from calcium and potassium channels. Specifically we used a caesium chloride based pipette solution (to minimise potassium currents) and Evans extracellular saline containing 50-100 μM cadmium chloride (to minimise calcium currents). In subsequent experiments where I_{NaP} was examined under more physiological conditions, a potassium-based internal solution (see above) was used and cadmium was excluded from the extracellular saline. In both scenarios linear 55 mV/s ramps were used so that transient sodium currents were inactivated and thus did not contaminate ramp traces. Pharmacological isolation of I_{NaP} was achieved by application of VGSC blockers tetrodotoxin (TTX, 0.01-1 μM) or riluzole (5-10 μM). For Boltzmann analysis individual current traces were leak subtracted prior to averaging. Estimates of membrane resistance were calculated by determining the slope of the V-I relationship in ohmic regions of the curve (*ca*. -80 mV). To monitor chloride and cation currents during locomotor activity, reversal potential of ionic species was determined experimentally by monitoring spontaneous currents at various holding potentials prior to stimulation of motor activity.

Current-clamp recordings were carried out to study spontaneous network activity, neuronal firing properties and locomotor drive. Prior to recordings, neurons were routinely bridge-balanced using the electronic features of the amplifier. When studying neuronal firing properties, the synaptic blockers strychnine (1 μM), bicuculline (10 μM) and kynurenic acid (2-4 mM) were included in the extracellular saline. To study rheobase (measured as the amount of injected current at action potential threshold), a small amount of holding current was injected into neurons to maintain a membrane potential of *ca*. -73 mV. From this potential depolarising current steps, ranging between -20 pA to 100 pA, were applied. For experiments in which firing properties of coiling stage embryos were examined, we used 26-29 hpf embryos as in earlier embryos neurons received regular driving inputs from gap junction coupled neighbours. As gap junctions are resistant to pharmacological blockade [9, 10] (H. Tong, unpublished observations), it was difficult synaptically isolate neurons and thus separate intrinsic activity from network-driven events. This problem was ameliorated by recording from late stage coiling fish (26 – 29 hpf) as here network activity is infrequent.

Simultaneous patch clamp recordings were performed as previously described [3, 8]. Briefly recordings were obtained from an IC cell in the rostral spinal cord and an ipsilateral CPG neuron located 1–3 spinal segments caudal to it. Upon completion of experiments neuronal identities were determined via fluorescence detection of sulforhodamine labelling.

During experiments in which fictive swimming was monitored, locomotion was triggered via application of a small amount of current delivered to the tail skin using a stimulating electrode connected to a stimulus isolation unit (Digitimer, UK).

Drugs

All drugs were obtained from Sigma (UK) with the exception of TTX, riluzole, apamin, ZD7288 and kynurenic acid which were obtained from Ascent Scientific (UK). Stock solutions were made in water or DMSO and diluted in Evans to final working concentrations. To study pharmacology of network activity, drugs were added directly to the perfusion saline. During voltage and current clamp studies of cellular currents and firing properties, drugs were applied focally using a gravity-fed VC3-4 perfusion system (ALA Scientific).

Electrophysiological analysis

PD durations were assessed from the average of 10-20 consecutive events obtained from each experiment. The difference between resting membrane potential and steady stage voltage at PD peak was used to assess amplitudes. To determine PD frequencies the number of events that occurred over a 100 s was calculated. Estimates of baseline membrane potential were derived from quiescent periods of recordings. However, in instances where SNA was high, this value was determined after silencing of network activity with 1 μM TTX. EPSP parameters were determined from the average of 10-30 events obtained from each locomotor-related episode and amplitude of tonic depolarisation was measured as the mean steady-state depolarisation from each recorded swim episode.

Because I_{NaP} appeared identical in all recorded cells, voltage clamp data was combined for generation of mean IV and conductance plots. Persistent sodium conductances were calculated from averaged

I_{NaP} by taking account of the driving forces, and fitted with a Boltzmann function; $g/gmax = (1+exp(V-V1/2/K))-1$ where g and gmax are conductances at membrane potential (V) and the maximum conductance, respectively, V1/2 is the half-activation voltage and K is the slope factor.

Behavioural analysis

To assess the effects of ion channel blockers on coiling, 22-24 hpf embryos were embedded in an agarose-lined dish containing egg water. Their tails were cut free and, with the exception of riluzole which was added directly to the egg water, the desired drug or control saline was injected into the yolk sac using a fine glass needle. After a 90 min incubation, 1 minute sequences of activity were filmed with a video camera (Basler, Germany; acquisition rate of 210 Hz) connected to a PC running VirtualDub (www.virtualdub.org). Sequential frames were montaged or stacked in ImageJ for presentation.

To assess the effects of dopamine on swimming, control saline or dopamine (100 μM) was injected into the yolk sac of 42 hpf embryos which were then left to recover for 60 min. Then fish were transferred to a 35 mm Petri dish and free-swimming responses evoked by sensory stimuli (touching of the tail with a pair of fine forceps) were recorded as described above.

Statistics

All data are presented as mean ± s.e.m. and statistical significance determined using two-tailed Student's t tests. A p value of < 0.05 was considered statistically significant.

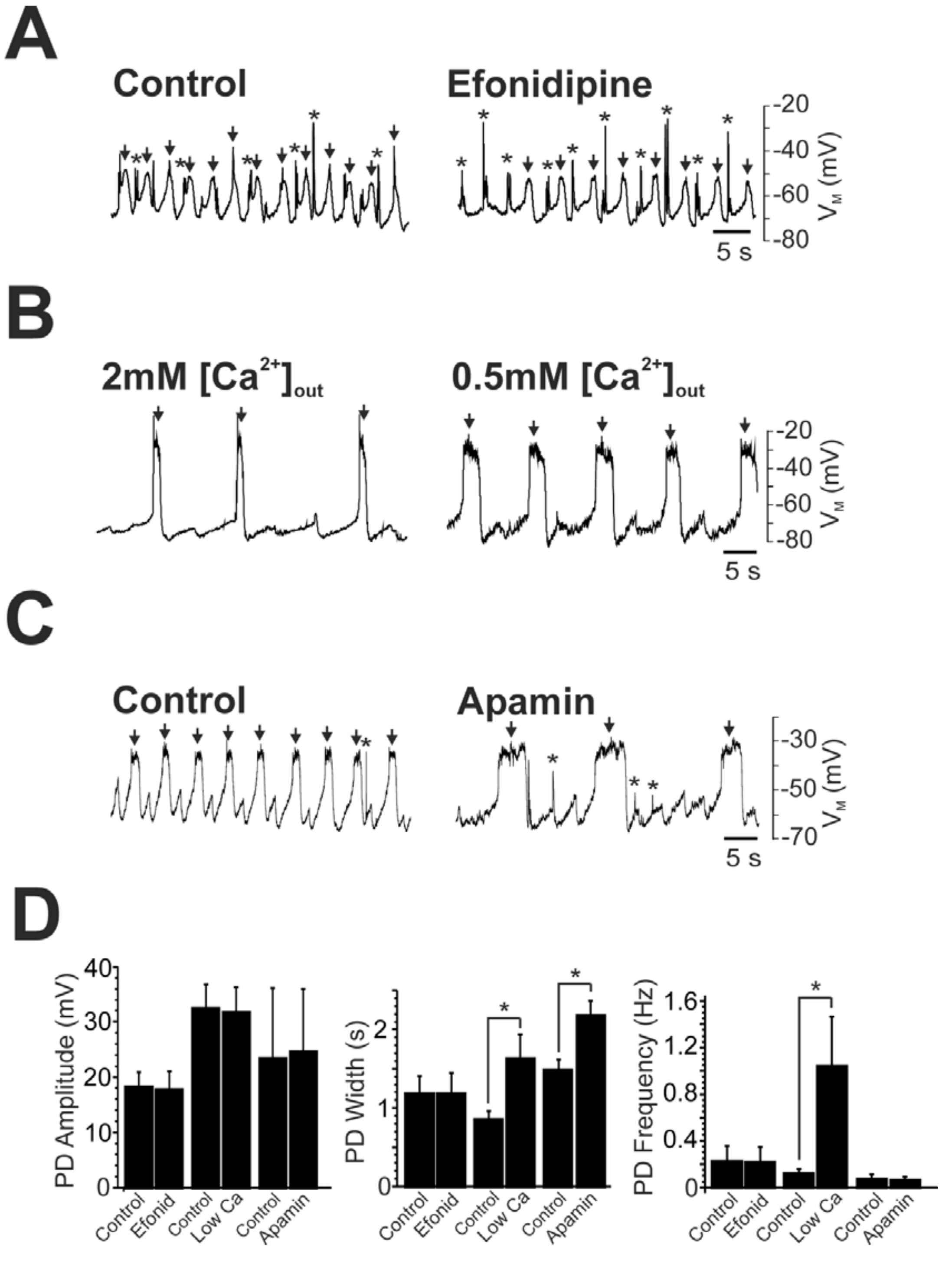

Figure S1. Role of Calcium during SNA, Related to Figure 2

(A,B) Representative voltage traces depicting effects of efonidipine (100 µM, **A**), low calcium (0.5 mM) saline (**B**) and apamin (40 nM, **C**) on SNA. Recordings were obtained from Mns (**A,B**) and VeLD interneurons (**C**) at 24, 25 and 24 hpf respectively. Arrows and asterisks show PD and SB, respectively. **(D)** Bar charts depicting effects of Efonid, low calcium saline (0.5–1 mM; Low Ca) and apamin on mean PD amplitude (left hand panel), width (middle panel) and frequency (right hand panel). Note that low calcium saline significantly increased PD duration (control saline = 0.86±0.10 s *cf.* low Ca saline = 1.64 ± 0.3 s, n = 8, *p = 0.009) and PD frequency (control saline = 0.12±0.01 Hz *cf.* low Ca saline = 1.04 ± 0.42 Hz, *p = 0.046) whilst apamin significantly increased PD duration (control = 1.49 ± 0.13 s *cf.* apamin 2.18 ± 0.19 s, *p = 4.5x10^{-3}). Data are represented as mean ± SEM.

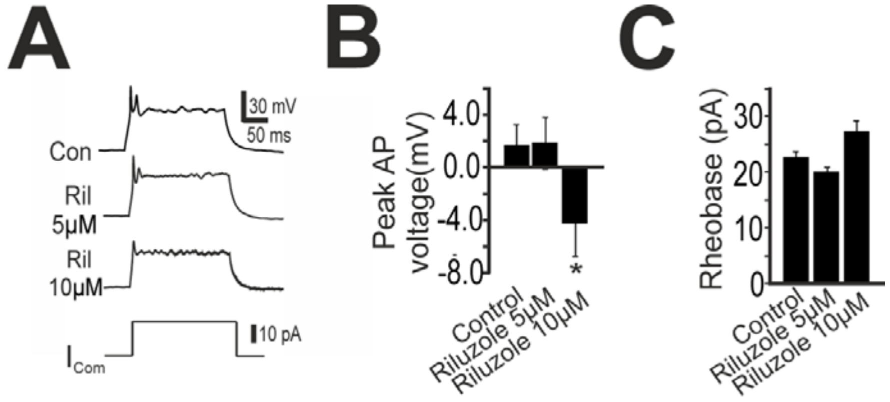

Figure S2. Effects of Riluzole on Action Potential Properties, Related to Figure 3
(A) Representative voltage recordings of Mn action potentials (APs) at 22 hpf, evoked by threshold current injection in control and in the presence of 5 µM and 10 µM riluzole respectively. **(B)** Bar chart depicting effects of riluzole on membrane voltage at peak AP amplitude. 5 µM riluzole (n=20) did not affect peak AP amplitude (p = 0.97) but 10 µM riluzole significantly reduced this parameter (n = 13 *p = 0.03). **(C)** Neither 5 µM (n = 20, p = 0.33) nor 10 µM (n = 13, p = 0.12) riluzole affected rheobase (i.e. current threshold for AP firing). Data are represented as mean ± SEM.

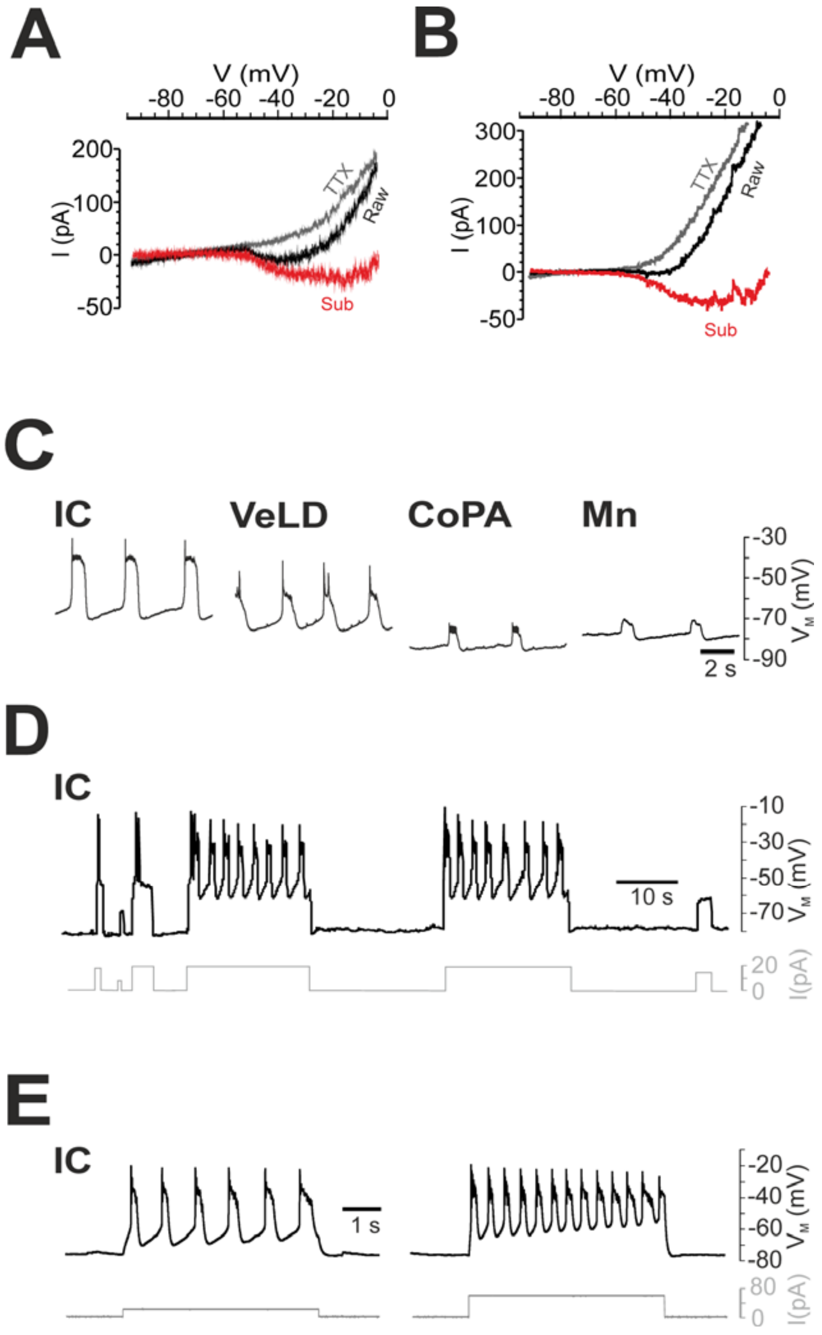

Figure S3. I_{NaP} Underpins IC Cell Oscillations at the Coiling Stage, Related to Figure 4
(A,B) Raw V-I traces (55mV/s ramps) of derived from an IC cell **(A)** and Mn **(B)** in control saline (black) and after addition of 1μM TTX (grey). Perfusion of TTX blocked inward rectification in the IC cell (**A**) and increased outward rectification in the Mn (**B**). Red traces depict TTX-sensitive subtraction current (sub). **(C)** Sample voltage recordings of each cell class at 22-23 hpf showing differences between PD amplitude and baseline membrane potential. **(D)** IC cell oscillations were conditional on depolarising input and terminated immediately upon release of command current. **(E)** Oscillation frequency was dependent on magnitude of current injection. 20 pA depolarising current evoked 1.5 Hz oscillations (left hand panel) whilst 60 pA evoked 2.2 Hz oscillations (right hand panel). Traces in **D-E** are derived from separate current clamp recordings at 28 and 27 hpf respectively.

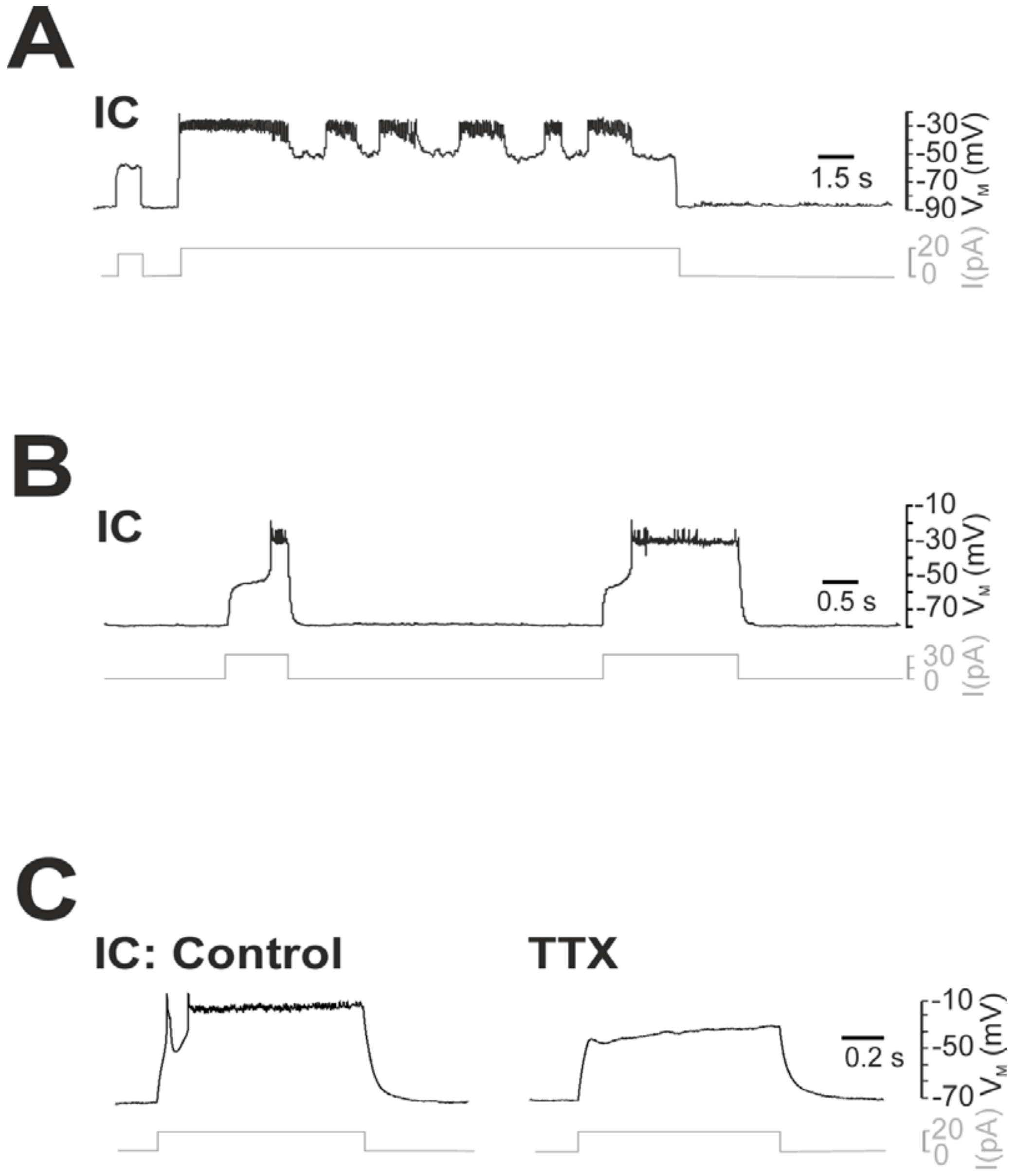

Figure S4. IC Cells and Sustained Bursts at the Early Swimming Stage, Related to Figure 5
(A) At early swimming stages IC cells often generated repetitive bursts in response to prolonged depolarising steps. **(B)** IC cell bursts were conditional on depolarising input because neurons immediately returned to rest upon termination of the current command. **(C)** During IC cell recordings, bath perfusion of TTX (1 μM) abolished both action potential generation and sustained bursts. Traces in **A**-**C** derived from separate current clamp recordings spanning 32-46 hpf. Current commands are depicted by grey traces.

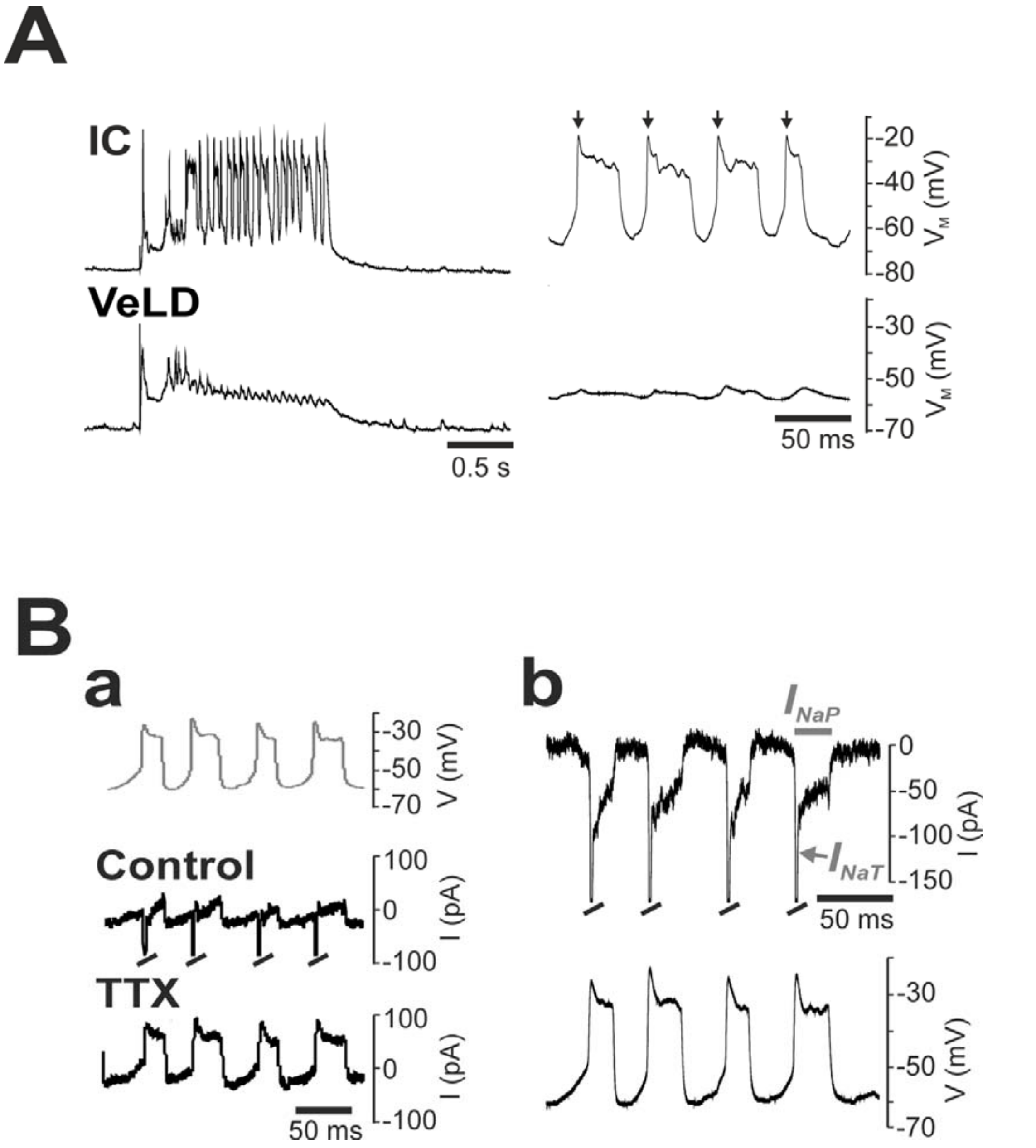

Figure S5. IC Cell Locomotor Drive during Early Swimming Periods, Related to Figure 6
(A) Left-hand panel: paired voltage recording of an IC cell and a VeLD interneuron 31 hpf. Right hand panel: Expanded sweep taken from corresponding episode showing rhythmic IC cell bursting occurring in register with rhythmic EPSPs in VeLD interneurons. Note that small spikelets (arrows) were often observed at the peak of IC cell bursts. **(B) (a)** IC cell current responses to a voltage waveform derived from an IC cell during fictive locomotion. Upper trace: command waveform. Middle trace: current response in control saline. Lower trace: current response after 8 min perfusion of TTX (1 μM). **(b)** Sodium current flow (upper trace, isolated by subtraction of current response in TTX from current response in control saline) during the voltage command (lower trace). Note that the TTX sensitive current comprises cyclically active persistent currents (presumably I_{NaP}) and large, transient currents (presumably I_{NaT}). I_{NaT} peaks in **a,b** have been cropped (oblique lines).

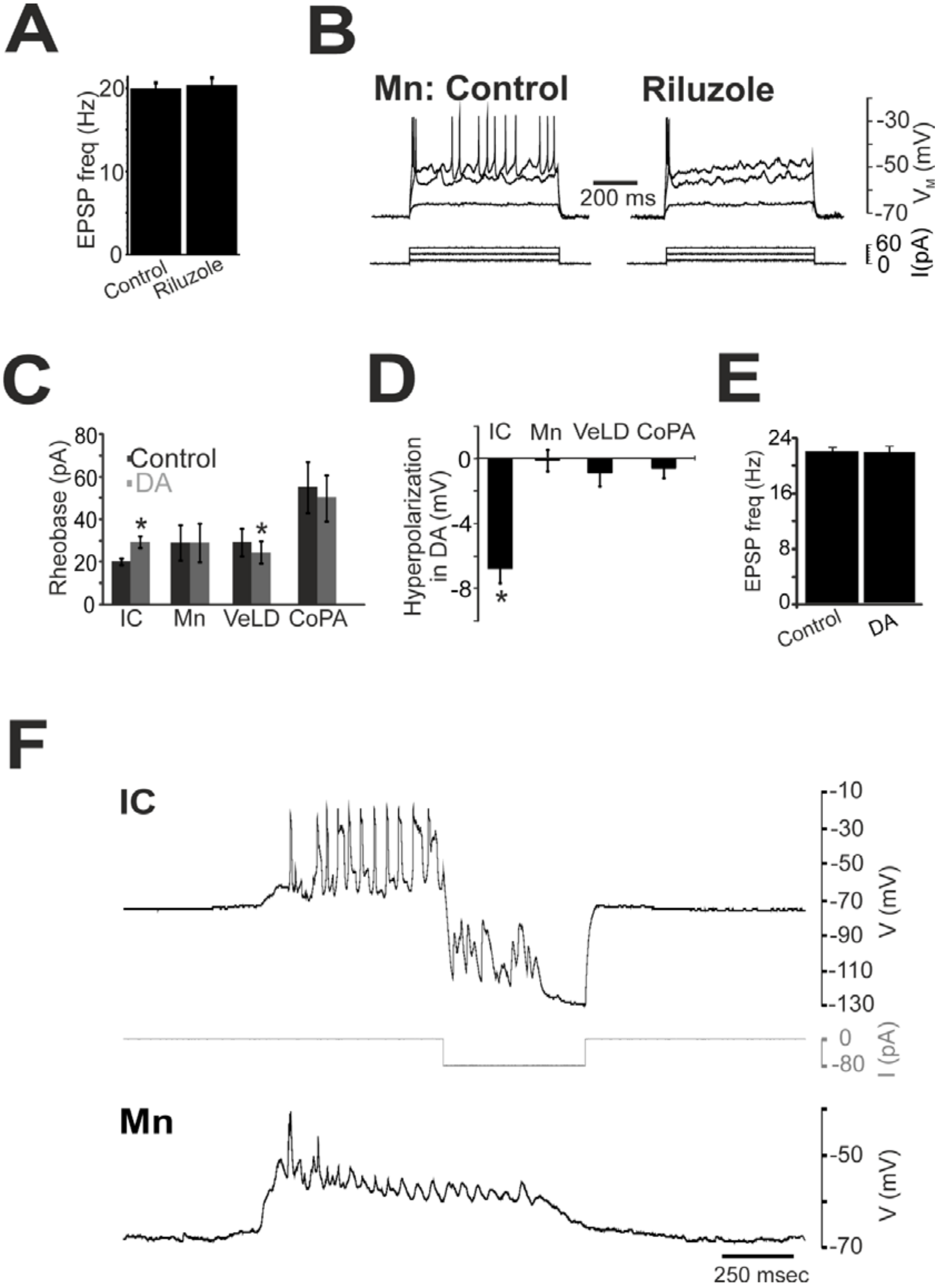

Figure S6. Effects of Riluzole, Dopamine and Hyperpolarisation on IC cells, Related to Figure 7
(A) Effects of riluzole on EPSP frequency during locomotor episodes recorded from primary Mns at 35–40 hpf. **(B)** Voltage responses (upper traces) to a family of current pulses (lower traces) recorded from a primary neuron at 38 hpf in control saline and after 10 min exposure to riluzole (5 µM). Note that riluzole abolishes repetitive spike capability in this cell. **(C)** Effects of dopamine (DA) on rheobase current of the four primary neurons studied. Dopamine increased IC cell firing threshold (control = 20.00 ± 1.54 pA *cf.* DA = 29.29 ± 2.77 pA, *p = 2×10^{-3}) and decreased VeLD threshold (control = 29.3 ± 5.7 *cf*. DA = 25 ± 4.6, *p = 0.048) but had no effect on other cell classes (**D**).

Changes in resting membrane potential of each primary neuron following dopamine (DA) perfusion. Dopamine hyperpolarised resting potential of IC cells (*p = $2x10^{-4}$) but had no effect on other cell classes Data in **C**,**D** obtained from embryos at 30-48 hpf. **(E)** Effects of dopamine (DA) on EPSP frequency during locomotor episodes recorded from primary Mns at 35–40 hpf. **(F)** Simultaneous recording of an IC cell (upper trace) and Mn (lower trace) during locomotor activity. Injecting the IC cell with a hyperpolarising current step (middle trace) does not impair Mn activity. Data in **A**,**C**-**E** represented as mean ± SEM.

SUPPLEMENTAL REFERENCES

1. Westerfield, M. (1994). The Zebrafish Book: a Guide to the Laboratory Use of the Zebrafish (Brachydanio rerio), 2.1 Edition, (University of Oregon Press, Eugene, OR).
2. Kimmel, C.B., Ballard, W.W., Kimmel, S.R., Ullmann, B., and Schilling, T.F. (1995). Stages of embryonic development of the zebrafish. Developmental Dynamics *203*, 253-310.
3. Drapeau, P., Ali, D.W., Buss, R.R., and Saint-Amant, L. (1999). In vivo recording from identifiable neurons of the locomotor network in the developing zebrafish. Journal of Neuroscience Methods *88*, 1-13.
4. Mendelson, B. (1986). Development of reticulospinal neurons of the zebrafish. II. Early axonal outgrowth and cell body position. Journal of Comparative Neurology *251*, 172-184.
5. Myers, P.Z., Eisen, J.S., and Westerfield, M. (1986). Development and axonal outgrowth of identified motoneurons in the zebrafish. Journal of Neuroscience *6*, 2278-2289.
6. Kuwada, J.Y., and Bernhardt, R.R. (1990). Axonal outgrowth by identified neurons in the spinal cord of zebrafish embryos. Experimental Neurology *109*, 29-34.
7. Bernhardt, R.R., Chitnis, A.B., Lindamer, L., and Kuwada, J.Y. (1990). Identification of spinal neurons in the embryonic and larval zebrafish. Journal of Comparative Neurology *302*, 603-616.
8. Saint-Amant, L., and Drapeau, P. (2003). Whole-cell patch-clamp recordings from identified spinal neurons in the zebrafish embryo. Methods in Cell Science *25*, 59-64.
